# Hypertrophic Cardiomyopathy and Wolff-Parkinson-White Syndrome in a Young African Soldier with Recurrent Syncope

**DOI:** 10.1155/2019/1061065

**Published:** 2019-12-04

**Authors:** Mohammed Abdullahi Talle, Faruk Buba, Aimé Bonny, Musa Mohammed Baba

**Affiliations:** ^1^Department of Medicine, College of Medical Sciences, University of Maiduguri, Nigeria; ^2^Cardiology Unit, Department of Medicine, University of Maiduguri Teaching Hospital, Maiduguri, Nigeria; ^3^University of Douala, Cameroon; ^4^Cardiology Unit, Department of Medicine, College of Medical Sciences, Gombe State University, Gombe, Nigeria

## Abstract

Syncope is a common manifestation of both hypertrophic cardiomyopathy (HCM) and Wolff-Parkinson-White (WPW) syndrome. The most common arrhythmia in HCM is ventricular tachycardia (VT) and atrial fibrillation (AF). While preexcitation provides the substrate for reentry and supraventricular tachycardia (SVT), AF is more common in patients with preexcitation than the general population. Concurrence of HCM and WPW has been reported in many cases, but whether the prognosis or severity of arrhythmia is different compared to the individual disorders remains unsettled. We report a case of HCM and Wolff-Parkinson-White (WPW) syndrome in a 28-year-old male Nigerian soldier presenting with recurrent syncope and lichen planus.

## 1. Introduction

Inherited cardiac arrhythmogenic disorders are less reported in Africa, and their burden as causes of sudden cardiac death (SCD) in Africa is unknown [1]. The lack of the often-needed expensive state-of-the-art diagnostic equipment compounds the challenges that hinder establishing the epidemiology of cardiac arrhythmias in sub-Saharan African (SSA) countries [2]. This makes the reporting of every single case of clinical arrhythmia (symptomatic or otherwise) imperative, to aid in better understanding of the burden of arrhythmic cardiac disorders in SSA.

Concurrence of HCM and WPW has been variously reported. In a consecutive series of patients presenting with preexcitation, 7.62% were found to have HCM [3]. Of the many phenocopies of HCM, cardiac hypertrophy and preexcitation are typically caused by a mutation of the gamma 2 subunit of the adenosine monophosphate-activated protein kinase (PRKAG2) [4]. Although both HCM and WPW are independently associated with various forms of arrhythmias and SCD, whether their concurrence will result in more frequent and severe arrhythmias or fatalities remains conjectural, and to our knowledge, this has not been reported in SSA. We present a case of HCM with WPW in a young African soldier presenting with recurrent syncope.

## 2. Case Presentation

A 28-year-old male African soldier from Nigeria was referred to the cardiology clinic of the University of Maiduguri Teaching Hospital in November 2017 for evaluation of recurrent syncope. He complained of paroxysmal palpitations, dizziness, and shortness of breath followed by presyncope and syncope. He has had subtle and infrequent palpitation and shortness of breath since childhood. He experienced his first syncope about 2 years ago which became more frequent three months prior to his referral and has had about 10 episodes of syncope. The last episode was in November 2017, during which a heart rate of 184 beats per minute and blood pressure of 90/50 mmHg with cold clammy extremities were documented by the attending medical officer at a peripheral hospital. However, ECG was not done due to nonavailability of ECG machine. There was no family history of similar symptoms or SCD. He developed pruritic skin lesions involving all parts of the body but mainly over the extensor surfaces a month prior to presentation. He joined the military 4 years ago after undergoing the rigorous recruit training program and has been actively involved in military operations until recently when his disabling symptoms precluded active participation.

**Figure 1:**
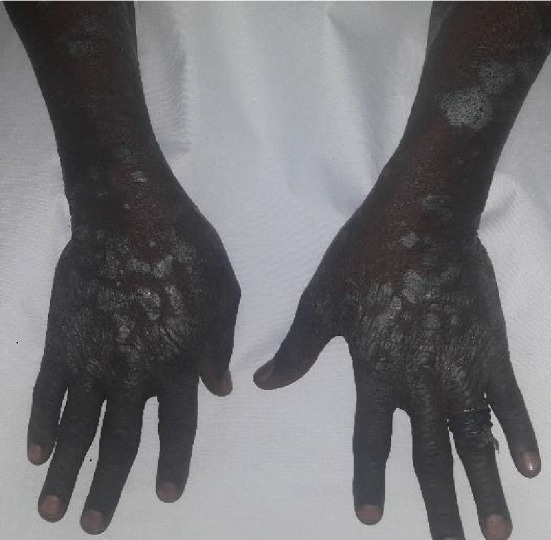
Silvery scaly lesions and plaques on the extensor surfaces of the hands and forearm.

The resting pulse was regular at 78 bpm, and all peripheral pulses were present and normal. He had a comparable blood pressure of 120/76 mmHg on the right and left upper limbs, and jugular venous pressure was normal. Apex was in the fifth left intercostal space at the midclavicular line and was heaving with double apical impulse. The heart sounds were S_4_, S_1_, and S_2_ with a crescendo-decrescendo systolic murmur loudest midway between the apex and the left lower sternal edge, but there was no diastolic murmur. Chest was clear, and no abnormality was detected on abdominal examination. Nervous and musculoskeletal systems were essentially normal. There were widespread silvery scaly skin lesions interspersed with hyperpigmented plaques all over his body but mainly on the extensor surfaces of the upper limb (Figure 1).

**Figure 2:**
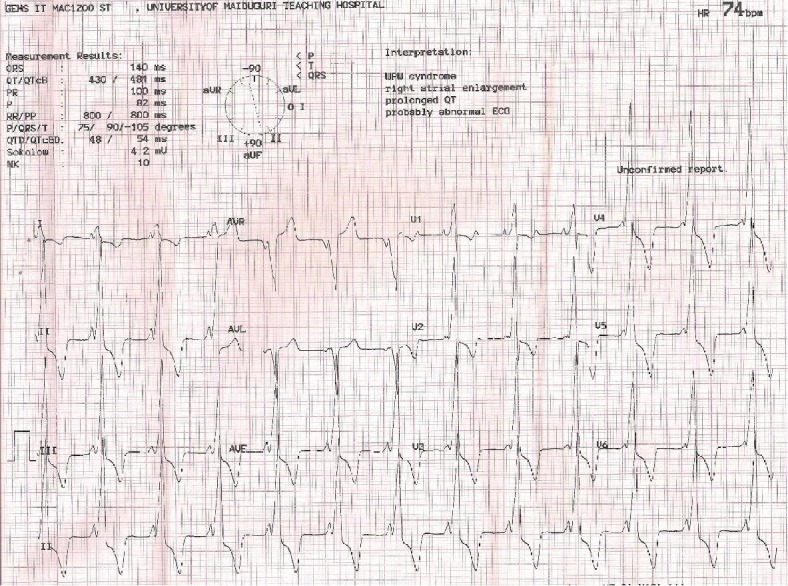
Resting 12-lead ECG obtained at presentation showing short PR interval, positive delta wave in V1 (type A Wolff-Parkinson-White pattern), and broad QRS complex.

Resting ECG (Figure 2) revealed a regular sinus rhythm at the rate of 78 cycles per minute with a QRS axis of +90°. The PR interval was about 100 msec, and there was a delta wave with broad QRS complex; findings are consistent with ventricular preexcitation. The QRS complexes showed positive concordance across precordial leads, with discordant ST segments and T waves. Corrected QT interval (adjusted for the broad QSR complexes) was prolonged at 461 msec.

**Figure 3:**
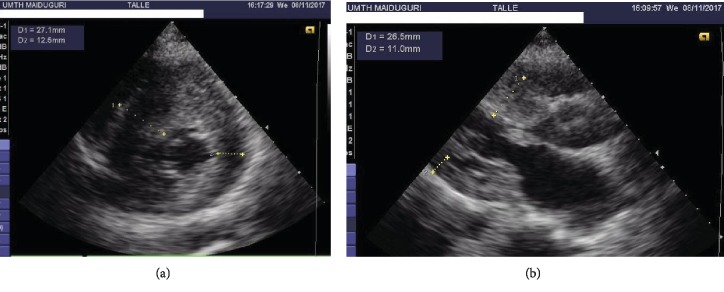
Parasternal short axis (a) and long axis (b) views showing asymmetric septal hypertrophy.

**Figure 4:**
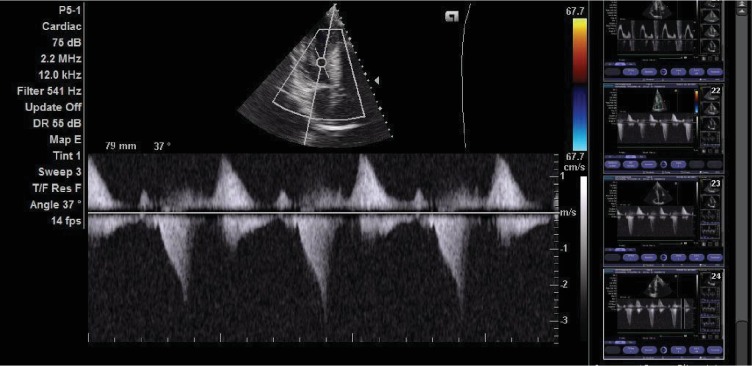
Late peaking of flow (dagger shape) across the left ventricular outflow tract.

Two-dimensional transthoracic echocardiography revealed a maximum septal thickness of 27 mm at the basal anterior septum with a corresponding posterior wall thickness of 13 mm (Figures 3(a) and 3(b)). Left ventricular outflow peak gradient was 36 mmHg, with a late peaking of velocity (Figure 4). There was no systolic anterior motion of the mitral valve or mitral regurgitation. The left ventricular end-diastolic dimension and ejection fraction were 21 mm and 80%, respectively. Exercise stress test was aborted within the first 2 minutes due to dizziness. Stress echo, cardiac MRI, cardiac CT, catheterization, ambulatory ECG, and electrophysiology (EP) study were not available.

A diagnosis of HCM with WPW was made, and he was placed on 100 mg of atenolol daily. A medical report was given to his division advising against strenuous activities and the need for further evaluation with ambulatory ECG and EP study. His regular follow-ups were uneventful, and he was recently transferred to the southern part of Nigeria where he will continue his treatment. His skin lesion was reviewed by a dermatologist, and a clinical diagnosis of lichen planus was made and confirmed by histology at another hospital. The third author (AB) offered to sponsor the subsequent evaluation and management of the patient in France or Algeria, but the patient declined the offer.

## 3. Discussion

This to the best of our knowledge is the first case of concurrence of HCM and WPW being reported from SSA. Both HCM and WPW can independently result in the constellation of symptoms our patient presented with. Hypertrophic cardiomyopathy has a protean manifestation, being generally asymptomatic or minimally symptomatic with a relatively benign course and annual mortality of less than 1% in the adult population. Clinical symptoms include progressive shortness of breath, chest pain, palpitations, light-headedness, syncope, and less commonly SCD. Palpitation in HCM results from supraventricular arrhythmias, commonly AF, and nonsustained ventricular tachycardia [5, 6]. The actual cause of recurrent palpitation and syncope in the index case was not determined because he did present to us while the symptomatic and ambulatory ECG and EP study was not done.

Wolff-Parkinson-White syndrome is an accessory pathway-mediated tachycardia occurring in patients with ventricular preexcitation and is the second most common cause of SVT [7]. Symptoms commonly include palpitations, shortness of breath, presyncope, syncope, and SCD. Thirty percent of people with WPW have AF, and this is associated with increased risk of SCD [8]. The patient reported 10 episodes of syncope in the preceding two years, and all were associated with palpitation. Although none of the episodes was captured on ECG, the last episode of witnessed event with regular pulse of 184 beats per minute before his referral is suggestive of SVT rather than AF. Rapid AF can be swiftly conducted to the ventricle via the accessory pathway resulting in 1 : 1 conduction and ventricular fibrillation with circulatory collapse. Whether the patient's syncopal episodes were associated with AF remains undetermined.

The association of WPW and other primary cardiac disorders has been variously documented. Mutation of PRKAG2 results in familial WPW with HCM [4]. Our patient and his kindred were not subjected to genetic studies due to nonavailability. However, there was no history suggesting familial occurrence of WPW and/or HCM, and the nervous and musculoskeletal disorders as well as conduction system disorders prevalent in this mutation were not documented in this patient.

Resting 12-lead ECG showed features of preexcitation, which along with recurrent palpitations and syncope defines WPW syndrome. The positive delta wave in V1 (type A WPW) and positively concordant QRS complexes across precordial leads are suggestive of the left lateral accessory pathway. However, EP study is required to confirm location and culpability of the pathway in cardiac arrhythmias but was not done due to nonavailability. Although the amplitude of the QRS complexes is in keeping with left ventricular hypertrophy (LVH), voltage criteria for ECG diagnosis of LVH may not be applicable in the setting of preexcitation, especially in persons younger than 40 years. The discordance of ST segments and T wave to the dominant deflection of QRS complexes in this context is more in keeping with ventricular preexcitation than LVH.

The diagnostic hallmark for HCM on echocardiography is asymmetric septal hypertrophy with a septum to posterior wall thickness ratio of greater than 1.5 or 1.3 in people with a family history of HCM. Our patient had asymmetric septal hypertrophy with a septum to posterior wall ratio of 2.5 and resting LVOT gradient of 36 mmHg. The military background of the patient makes physiologic LVH (athleticism) a pertinent consideration. However, the septal thickness of 27 mm is well out of the “grey zone” of 13 to 15 mm that commonly results in such diagnostic challenges. Similarly, the presence of restrictive LV filling pattern on spectral Doppler suggesting diastolic dysfunction and limited left ventricular end-diastolic dimension favours HCM.

Whether this patient's recurrent syncope was a consequence of arrhythmias related to either WPW or HCM was not established with certainty. Notwithstanding, recurrence of syncope in this context puts the patient at a very high risk and requires further evaluation and implementation of definitive treatment strategies to avert SCD. Determining the culprit for the recurrent syncope between WPW and HCM presents a real diagnostic conundrum.

Ambulatory ECG and EP study would have provided insights. The frequency of syncope remarkably improved on 100 mg of atenolol, and the patient had only one episode in four months.

The diagnosis of lichen planus may add another layer of intrigue to the case. An association has been established between dermatologic disorders (e.g., psoriasis) and cardiovascular diseases, reflecting the role of inflammation as a common denominator. However, no such link has been established between Lichen planus and WPW and/or HCM. Hypertrophic cardiomyopathy was reported in a 59-year-old woman with lichen planus and rheumatoid arthritis receiving etanercept and methotrexate [9]. Similarly, adalimumab (Humira), a tumour necrosis factor blocker used in the treatment of psoriatic arthritis, has been associated with HCM [10]. Skin manifestation is an integral part of Fabry's disease, a storage disorder having identical phenotype with HCM, but the appearance is different from the lesions in this patient.

The evaluation and management of this patient was limited by lack of facilities and the requisite expertise. Ambulatory ECG, EP study, cardiac MRI, and genetic testing would have undoubtedly provided pertinent information that will fine-tune the management but were not available. Consequently, decisions regarding ablation therapy and/or implantable cardioverter-defibrillator could not be accomplished. This case exemplifies the prevailing practices in many countries of sub-Saharan Africa, especially in management of cardiac arrhythmias.
